# Women’s participation in household decision-making and higher dietary diversity: findings from nationally representative data from Ghana

**DOI:** 10.1186/s41043-016-0053-1

**Published:** 2016-05-31

**Authors:** Dickson A. Amugsi, Anna Lartey, Elizabeth Kimani-Murage, Blessing U. Mberu

**Affiliations:** 1 African Population and Health Research Centre, APHRC Campus, P.O. Box 10787-00100, Nairobi, Kenya; 2 Nutrition Division, Economic and Social Department, Food and Agriculture Organization, Rome, Italy

**Keywords:** Women, Higher, Dietary diversity, Ghana

## Abstract

**Background:**

Low-quality monotonous diet is a major problem confronting resource-constrained settings across the world. Starchy staple foods dominate the diets in these settings. This places the population, especially women of reproductive age, at a risk of micronutrients deficiencies. This study seeks to examine the association between women’s decision-making autonomy and women’s achievement of higher dietary diversity (DD) and determine the socio-demographic factors that can independently predict women’s attainment of higher DD.

**Methods:**

The study used data from the 2008 Ghana Demographic and Health Survey. The participants comprised of 2262 women aged 15–49 years and who have complete dietary data. The DD score was derived from a 24-h recall of intake of foods from nine groups. The score was dichotomized into lower DD (DD ≤4) and higher (DD ≥5). Logistic regression was used to assess the association between women decision-making autonomy (final say on how to spend money, making household purchases, own health care, opinions on wife-beating, and sexual intercourse with husband) and the achievement of higher DD. The logistic regression models were adjusted for covariates at the individual and household levels.

**Results:**

The analysis showed that women participation in decision-making regarding household purchases was significantly associated with higher DD, after adjusting for individual and household level covariates. The odds of achieving higher DD were higher among women who had a say in deciding household purchases, compared to women who did not have a say (OR = 1.74, 95 % CI = 1.24, 2.42). Women who had more than primary education were 1.6 times more likely to achieve higher DD, compared to those with no education (95 % CI = 1.12, 2.20). Compared to women who lived in polygamous households, those who lived in monogamous households had higher odds of achieving higher DD (OR = 1.42, 95 % CI = 1.04, 1.93).

**Conclusions:**

Net other covariates, women who have a say in making household purchases are more likely to achieve higher DD compare to those who do not have a say. This may indicate autonomy to buy nutritious foods, suggesting that improving women decision-making autonomy could have a positive impact on women dietary intake.

## Background

Low-quality monotonous diet is the bane of resource constrained settings across the world [1]. Starchy staple foods dominate the diets in these settings, with fruits, vegetables, and animal source foods scarcely consumed. This places the population at high risk of micronutrient deficiencies, and women of reproductive age are particularly vulnerable [1–4]. This is the case because of increased nutrient needs for women during pregnancy and lactation, and when these needs are not met, mothers may experience wasting and fatigue that may limit their ability to fully satisfy infant needs [3]. This may result in infants who are small for gestational age and children with stunted growth and slowed cognitive development, which may persist into adulthood and transmit to the next generation [3]. Thus, the consequences of micronutrient malnutrition do not only affect the health and survival of women, but also their offspring. It is estimated that 2 billion people around the world suffer from micronutrient deficiency, and 19 million pregnant women are vitamin A deficient [2, 5]. The vast majority of these people live in the developing countries. There is a growing consensus that increasing the dietary diversity (more food groups) can help improve diet quality, especially among women [2, 3, 6]. A new dietary diversity indicator, called minimum dietary diversity-women (MDD-W) has recently been developed as a proxy indicator of the micronutrient adequacy of the diet of women [6–8]. The proponents of this indicator posit that women consuming foods from five or more food groups out of ten have a greater likelihood of meeting their micronutrient needs than women consuming foods from fewer food groups [8].

There is evidence that women’s participation in household decision-making and ability to purchase food (an aspect of empowerment) is significantly associated with availability of diverse diet in the household [9]. This enforces the idea that women’s decision-making autonomy is an important aspect of women empowerment, as it relates to women’s dietary diversity and subsequently, better nutritional status. Various elements of women empowerment and disempowerment for that matter have been linked to lower or higher nutritional risks respectively. For instance, domestic abuse and sexual coercion have an impact on women’s nutrition. A study in South India observed that mothers who had endured domestic abuse and sexual coercion were at higher risk of poor nutrition, so did their children [10]. Also, a positive association between increases in women’s empowerment and improved nutrition outcomes has been documented and, that any actions leading to women’s disempowerment can result in adverse nutritional impacts for women [11–13]. A study in Bangladesh and Vietnam observed a positive association between maternal education and maternal dietary diversity [14]. Women who have greater education tend to have higher dietary diversity (DD). Murakami and colleagues [15] investigated the association between maternal education and individual food consumption among Japanese women. They observed that higher education is associated with a higher intake of vegetables, fish and shellfish, and potatoes, but not with bread, noodles, confectioneries and sugars, fats and oils, pulses and nuts, meat, eggs, dairy products, or fruits [15]. Mirmiran and colleagues [16] also found significant positive association between educational level and improvement in women’s dietary intake.

A number of studies have found a significant association between Socio-economic status (SES) and women’s dietary diversity [16, 17]. A study in Nepal concludes that higher SES is associated significantly with more frequent consumption of most food groups, including in-season fruits and vegetables [17]. Other factors such as place of residence, presence of co-wives in the household and sex of household head have been found to associate importantly with women eating patterns [18–20]. For example, systematic review of studies on dietary patterns in low- and middle-income countries reveals that living in urban areas is associated with overall healthier dietary patterns [18].

The literature reviewed above helps one appreciate the significance of women’s decision-making autonomy in promoting women’s DD. However, the challenge with the foregoing literature is the glaring lack of evidence on African countries. In Ghana for instance, studies on how women participation in decision-making at the household level impacts on their dietary diversity is almost non-existent. This highlights the need to examine data from Ghana on the subject of decision-making and DD. The present study was therefore set out to (1) examine the association between women’s decision-making autonomy and women’s achievement of higher dietary diversity (DD); (2) determine the socio-demographic factors that can independently predict women’s attainment of higher DD.

## Methods

### Data sources and study population

This analysis used data from the Ghana Demographic and Health Surveys (GDHS) [21]. The surveys were conducted in Ghana in 2008 (September to November) by the Ghana Statistical Service and the Ghana Health Service, with technical support from ICF Macro through the MEASURE DHS programme. The surveys were designed to be representative at the national, regional and rural-urban levels. The Ghana DHS employed a two-stage sampling design. The first stage involved selection of clusters from a master sampling frame constructed from the 2000 national population and housing census. The second stage involved the selection of households from these clusters. All women and men aged 15–49 and 15–59, respectively, in the selected households were eligible to participate in the survey. Three questionnaires were used for the data collection: the Household Questionnaire, the Women’s Questionnaire, and the Men’s Questionnaire. The household response rate was 98.9 %. In this analysis, we used data of 2262 women aged 15–49 years and who have complete dietary data. Ethical clearance was sought from Ghana Health Service ethics review committee (GHS-ERC) before the surveys were conducted. Individual written informed consent was obtained from study participants before they were allowed to participate in the study. The data were completely anonymous; therefore, the authors of the present study did not have to seek further ethical clearance.

### Measures

#### Women’s dietary diversity

The women’s DD score was constructed based on the 24-h recall of food consumption. The DD score is a count of the number of food groups consumed by the woman during the 24 h prior to the in-home survey. Fourteen (14) types of foods contained in the data were regrouped into nine (9) main food groups [3]: (1) grain, tubers, roots; (2) flesh meat (beef, pork, chicken, fish etc.); (3) dairy products (milk, cheese, yogurt etc.); (4) legumes (food made from beans, peas, lentils, nuts); (5) eggs; (6) organ meat (liver, heart, kidney etc.); (7) dark green vitamin A rich leafy vegetables; (8) vitamin A rich fruits and other vitamin A vegetables; (9) other fruits. The women reported whether or not they have consumed any of the above mentioned food groups. A “yes” response was scored “1” and “no” response scored “0”. The scores were then summed up to create the women DD score, which ranged from 0 to 9. This score was dichotomised into consumption of four or less food groups (DD ≤4)—lower DD, and consumption of five or more food groups (DD ≥5)—higher DD, and used in the subsequent analysis. The selection of higher DD threshold of five (5) food groups was informed by the new recommended dietary diversity indicator for women, called minimum dietary diversity-women (MDD-W), for assessing micronutrient adequacy for women’s diets [6]. This indicator reflects consumption of at least five of ten food groups. However, because the food groups used in this paper were not the actual representation of the MDD-W indicator, the use of the term MDD-W had been avoided.

#### Women’s decision-making autonomy (empowerment) variables

Women empowerment variables used in this analysis included, final say on how to spend money, final say in making household purchases, final say on own health care, opinion on wife-beating, and ability to refuse husband sex on justifiable grounds. Some of the variables were recoded. Final say on making large household purchases and final say on making purchases for daily needs were recoded into “final say on making household purchases”. Also, final say on deciding what to do with money husband earns and who decides how to spend money in the household were recoded into “final say on how to spend money”. In addition, two indices on women autonomy were created [22]: the number of reasons that justify wife-beating in the respondent’s opinion and the respondent’s opinion on the number of circumstances under which a wife is justified in refusing to have sexual intercourse with her husband. Details on the construction of these indices have been published elsewhere [23].

#### Socio-demographic factors

These factors were collected both at the woman and household levels. The woman level factors included but are not limited to age, education, parity, occupation, ethnicity, and religion. The household level factors included sex of household head, place of residence, household wealth index, presence of co-wives, number of children under 5 years, and number of household members. Ethnicity and religion were recoded into “Akan” and other ethnicities” and “Christian and other religions”, respectively. The occupation variable was also recoded into “white collar and agriculture/labour”.

### Statistical analysis

The data were analysed using IBM SPSS version 21. Descriptive analysis was conducted to examine the background characteristics of the study samples. Bivariate analysis was carried out to assess the association between the predictor variables and higher women’s DD. Only significant predictors in bivariate analysis were used in the multivariate analysis. Logistic regression was used to assess the association between women decision-making autonomy (final say on how to spend money, making household purchases, own health care, opinions on wife-beating, and sexual intercourse with husband), and the achievement of higher DD. The logistic regression models were adjusted for covariates at the individual level (age, parity, education, occupation, ethnicity and religion), and household level (sex of household head, place of residence, wealth index, co-wives, number of children under 5 years and number of household members). Results were considered statistically significant when *P* < .05.

## Results

### Descriptive analysis results

Table 1 presents descriptive statistics of the sample. The average age of the study participants was 30 years (SD = 7.02). Twenty-one percent (21 %) of the study sample lived in polygamous households, and each household in the sample had approximately six members. Expectedly, almost all the women (98 %) in this sample consumed starchy food (grains, tubers, roots). The second highest consumed food group was flesh meat (82 %). The least consumed food groups were organ meat (10 %) and vitamin A rich fruits and other vitamin A vegetables (16 %). Additionally, 63 % of the women consumed non-vitamin A rich fruits (other fruits) while a total of 43 % consumed foods from five or more food groups.

**Table 1:** Descriptive analysis of the study sample (*n* = 2262): categorical and continuous variables

Variables	Means ± SD/%
Women dietary diversity (DD)	
Women DD ≤4	56.9
Women DD ≥5	43.1
Food groups used in creating women DD	
Grains-tubers-roots (yes)	97.9
Flesh food (beef, pork, fish etc.) (yes)	82.2
Dairy products (milk, cheese, yogurt etc.)(yes)	58.1
Legumes (food made from beans, peas, lentils, nuts) (yes)	28.4
Eggs (yes)	19.2
Organ meat (yes)	10.2
Dark green vitamin A rich leafy vegetables (yes)	55.6
Vitamin A rich fruits and other vitamin A vegetables (yes)	16.1
Other fruits (yes)	63.0
Socio-demographic factors	
Maternal occupation	
Agriculture/labour	54.2
White collar	45.8
Maternal education	
No education	37.8
Primary	24.1
Secondary+	38.0
Final say on own health care	
Has a say	65.2
Final say in making household purchases	
Has a say in taking final decision	79.2
Presence of co-wives in household	
There are co-wives	21.0
There are no co-wives	79.0
Continuous variables	
Maternal age	30.10 ± 7.02
Maternal parity	3.70 ± 2.24
Number of children under 5 years	1.78 ± 0.99
Number of household members	5.88 ± 2.76

### Bivariate analysis results

In the bivariate analysis, the following decision-making autonomy variables were positively and significantly associated with higher DD among women: final say on household purchases (β = 0.56, *P* = .001), final say on how to spend money (β = 0.51, *P* = .026), women opinion on wife-beating (β = 0.07, *P* = .022), and ability to refuse husband sex on justifiable grounds (β = 0.11, *P* = .043). Additionally, socio-demographic variables such as woman age, parity, education, occupation, ethnicity, religion, sex of household head, household wealth index, presence of co-wives, and number of household members were also significantly associated with higher women’s DD.

### Multivariate analysis results

Table 2 presents the results of the multiple logistic regression analysis. In this analysis, women decision-making autonomy regarding household purchases was significantly associated with higher DD (consumption of five or more food groups), after adjusting for individual and household level covariates. Compared with women who have no say in deciding household purchases, women who had a say were almost twice as likely to achieve higher DD (OR = 1.74, 95 % CI = 1.24, 2.42). The other decision-making variables (final say on how to spend money, women’s opinion on wife-beating, ability to refuse husband sex on justifiable grounds) did not reach statistical significance in the multivariate analysis. Women educational level was also associated with higher DD—women who had higher than primary education were 1.6 times more likely to achieve higher DD than those with no education (95 % CI = 1.12, 2.20). Compared to women in the poorest wealth quintile, women in the richer and richest quintiles were 1.7 (95 % CI = 1.07, 2.64) and 1.8 (95 % CI = 1.05, 3.14) times respectively, more likely to achieve higher DD. Women who live in non-polygamous households had higher odds of achieving higher DD than those who lived in polygamous households (OR = 1.42, 95 % CI = 1.04, 1.93). There were significant negative associations between Christian religion and Akan ethnic group, and higher DD respectively.

**Table 2:** Multivariate logistic regression analysis of the association between women’s decision-making autonomy, socio-demographic factors, and women’s dietary diversity (DD)^a^ (*n* = 2262)

Variables	Coefficients	SE	OR	95 % CI for OR	Wald F	*P*
Women decision-making autonomy						
Final say on how to spend money						
Has no say in making final decision	Ref	Ref	Ref	Ref	Ref	Ref
Has a say in making final decision	0.32	0.25	1.38	0.84, 2.25	1.63	0.200
Final say on making Household purchases						
Has no say in making final decision	Ref	Ref	Ref	Ref	Ref	Ref
Has a say in making final decision	0.55	0.17	1.74	1.24, 2.42	10.53	.001
Final say on own health care						
Has no say in making final decision	Ref	Ref	Ref	Ref	Ref	Ref
Has a say in making final decision	−0.25	0.13	0.78	0.60, 1.01	3.63	.057
Wife-beating not justified score	−0.01	0.04	0.99	0.92, 1.07	0.02	.900
Refused husband sex score	0.07	0.07	1.07	0.94, 1.23	1.03	.310
Woman level factors^b^						
Maternal age	0.01	0.01	1.01	0.98, 1.04	0.40	.530
Maternal parity	−0.04	0.05	0.96	0.88, 1.05	0.87	.350
Maternal education						
No education	Ref	Ref	Ref	Ref	Ref	Ref
Primary	0.17	0.16	1.18	0.86, 1.62	1.06	.300
Secondary or higher	0.45	0.17	1.57	1.12, 2.20	6.87	.009
Maternal occupation						
Agriculture/labour	Ref	Ref	Ref	Ref	Ref	Ref
White collar	−0.11	0.14	0.90	0.69, 1.17	0.66	.420
Maternal ethnicity						
Other ethnicities	Ref	Ref	Ref	Ref	Ref	Ref
Akan	−0.36	0.14	0.70	0.53, 0.91	7.11	.008
Maternal religion						
Other religions	Ref	Ref	Ref	Ref	Ref	Ref
Christian religion	−0.30	0.14	0.75	0.56, 0.99	4.18	.041
Household level factors^b^						
Sex of household head						
Male	Ref	Ref	Ref	Ref	Ref	Ref
Female	−0.18	0.14	0.84	0.63, 1.11	1.59	.210
Place of residence						
Rural	Ref	Ref	Ref	Ref	Ref	Ref
Urban	0.10	0.17	1.11	0.80, 1.55	0.38	.540
Household wealth index						
Poorest	Ref	Ref	Ref	Ref	Ref	Ref
Poor	−0.34	0.18	0.71	0.51, 1.01	3.71	.054
Middle	−0.31	0.21	0.73	0.48, 1.10	2.23	.140
Richer	0.52	0.23	1.68	1.07, 2.64	4.98	.026
Richest	0.60	0.28	1.81	1.05, 3.14	4.49	.034
Present of co-wives in household						
There are co-wives	Ref	Ref	Ref	Ref	Ref	Ref
There are no co-wives	0.35	0.16	1.42	1.04, 1.93	4.86	.028
Number of under five children	0.03	0.08	1.03	0.89, 1.19	0.13	.720
Number of household members	0.04	0.03	1.04	0.98, 1.11	1.92	.170

^a^Dietary diversity (DD) is the number of food groups consumed over 24-h period

^b^Women and household levels factors were adjusted for in the multivariate analysis

## Discussion

The results of our analyses show a strong positive association between women’s participation in decision-making (indication of empowerment) regarding household purchases and higher DD. Women who participate in final decision-making are almost two times more likely to achieve higher DD compared to those who do not participate in household decision-making. This association remains after adjusting for other important socio-demographic determinants of women’s dietary diversity at the individual and household levels. These results suggest that women’s decision-making autonomy at the household level is crucial for the consumption of diverse diet. The findings of the present study are consistent with previous studies. In India and Bangladesh, women’s participation in household decision-making and ability to purchase food, have positive impact on the availability of diverse diet in the household, and consequently adequate dietary diversity intake among women and children [9, 24]. This suggests that programmes to improve women’s nutrition could focus on increasing women’s decision-making power. Similarly, several other studies report positive associations between women’s empowerment and dietary diversity [11, 13, 25]. These studies conclude that any actions that lead to women disempowerment can result in adverse nutritional impact for women as well as for their children. Hence, it stands to reason that investment in women empowerment would have a beneficial effect on women and their children [13, 26, 27]. It is evident from the foregoing that women’s participation in decision-making (an indicator of empowerment) associates importantly with women dietary intake, although the underlying mechanisms may be complex.

Our analysis also reveals a number of socio-demographic factors that are associated significantly with women’s DD. One of such factors is women’s education. The results show that high level of women’s education increases the likelihood of achieving higher DD. This is probably because women who have high education are likely to earn their own income, thereby becoming financially autonomous. Indeed, it is a documented fact that financial autonomy has a positive influence on women’s nutrition [28]. This is so, because higher financial autonomy gives women more negotiation powers with regards to food purchases [1, 13, 28]. Our findings are comparable with previous studies. In Vietnam and Bangladesh, positive and significant association was observed between maternal education and maternal dietary diversity [14]. Nevertheless, in Japan, the effect of education on the intake of individual foods is mixed. While higher education was associated with a higher intake of vegetables, fish and shellfish, and potatoes, there was no association between education and intake of bread, noodles, confectioneries and sugars, fats and oils, pulses and nuts, meat, eggs, dairy products, or fruit [15]. This implies that when dietary diversity is disaggregated, the influence of education may not be across all food groups. Further analysis could focus on both dietary diversity and the individual food groups.

Furthermore, household wealth (an indicator of socio-economic status) was found in this analysis to associate significantly with women’s DD. Women in the richest wealth quintile have higher odds of achieving higher DD compared to those in the poorest wealth quintiles. The probable explanation is that women in the richest quintile in Ghana are likely to have available to them, a disposable income and other resources. This may increase the ability of women in these households to make household purchases [9]. The importance of food purchasing ability on the availability of dietary diversity in households has been documented [9].The findings of the present study are in line with previous studies that examined the relationship between socio-economic status (SES) and women dietary intake. Mirmiran and colleagues [16] observed a positive and significant association between SES and women dietary diversity. Additionally, two other studies concluded that higher SES is associated importantly with more frequent consumption of most food groups including seasonal fruits and vegetables, diet quality, and diversity [17, 18]. Our analysis confirms the findings from previous studies that SES is an important determinant of women’s dietary intake. Interventions targeted at increasing women’s SES could increase their dietary diversity.

Similarly, this study shows that compared to women who live in polygamous households, women in non-polygamous (monogamous) households are more likely to achieve a higher DD. This suggests that non-existent of co-wives reduces competition on the limited resources in the household, thereby making women in the monogamous households to have access to a diverse diet. As there is an evidence that polygamy forces women to live in poverty by forcing them to share resources in the household and thus exacerbates the impoverishment of women by limiting their access to financial and other resources in the marriage [19]. Another plausible explanation is the relationship between monogamy and women’s autonomy, and the effect it has on women’s dietary diversity. Nigatu and colleagues [29] observed a positive association between monogamous marriage and women’s autonomy—women in monogamous marriages were more than three times likely to be autonomous compared to those in polygamous marriages’. A related study observes that women in monogamous households have considerable decision-making power, including what the household will consume, while women in polygamous households have noticeable smaller decision-making power [30].

There are a number of strengths and weakness associated with this study. One of the key strengths is the representative nature of the data used in the analysis. This means that the findings of the study can be generalised to all women in Ghana. To the best of the authors’ knowledge, this is the first study in Ghana to have used nationally representative data to investigate the relationship between women’s decision-making autonomy, socio-demographic factors, and women’s DD.

A limitation of this study is the fact that the data are from a cross sectional study, and a causal relationship between socio-demographic factors, decision-making autonomy, and higher DD cannot be established. Therefore, the conclusions contained in this paper are based on associations between the explanatory and outcome variables, rather than causal relationships. Due to data limitation, the present study used 9-point food group instead of the newly recommended 10-point food group. Secondly, the present study included organ meat in the computation of the DD score, while the newly recommended MDD-W did not. However, since the DD use in this study is not intended to be an exact representation of the new MDD-W, and for the fact that the use of the term MDD-W had been avoided in the paper, the inclusion of organ meat might not affect the findings contained in this paper.

## Conclusions

The results show that women who participate in taking household decisions making regarding household purchases are more likely to achieve higher DD compared to those who do not participate. This suggests that improving women decision-making autonomy could have a positive impact on women’s dietary intake. Interventions in Ghana should target at promoting women’s decision-making power at the households level. Household wealth index, education, and the absence of co-wives in the household are also positively and significantly associated with higher women’s DD. Improving household wealth, promoting female education, and encouraging monogamous marriages could have a significant impact on women dietary intake in Ghana.
