# Erratum to: Women’s participation in household decision-making and higher dietary diversity: findings from nationally representative data from Ghana

**DOI:** 10.1186/s41043-016-0055-z

**Published:** 2016-06-16

**Authors:** Dickson A. Amugsi, Anna Lartey, Elizabeth Kimani-Murage, Blessing U. Mberu

**Affiliations:** 1 African Population and Health Research Centre, APHRC Campus, P.O. Box 10787-00100, Nairobi, Kenya; 2 Nutrition Division, Economic and Social Department, Food and Agriculture Organization, Rome, Italy

## Erratum

After publication of the original article [1] it was brought to our attention that one of the author’s names was not displaying correctly in the published version of the article. The name originally read as Elizabeth Kimani, whereas the name should read as Elizabeth Kimani-Murage. This has now been updated on the BMC website.

## References

[1] Amugsi (2016). Women’s participation in household decision-making and higher dietary diversity: findings from nationally representative data from Ghana. J Health Popul Nutr.

